# Evaluation of the Effect of Lymph Node Status on the Survival of Non-Small Cell Lung Cancer Patients With Brain Metastases: Applications of a Novel Grade Prognostic Assessment Score Model Involving N Stage

**DOI:** 10.3389/fonc.2020.563700

**Published:** 2020-10-19

**Authors:** Jianfei Zhu, Wuping Wang, Shuonan Xu, Chenghui Jia, Qingqing Zhang, Yanmin Xia, Wenchen Wang, Miaomiao Wen, Xuejiao Wang, Hongtao Wang, Zhipei Zhang, Ling Cai, Lanjun Zhang, Tao Jiang

**Affiliations:** ^1^ Department of Thoracic Surgery, Tangdu Hospital, Air Force Medical University (Fourth Military Medical University), Xi’an, China; ^2^ Department of Thoracic Surgery, Sun Yat-sen University Cancer Center, Guangzhou, China; ^3^ Department of Thoracic Surgery, Shaanxi Provincial People’s Hospital, Xi’an, China; ^4^ Department of Pulmonary and Critical Care Medicine, The First Affiliated Hospital of Xi’an Medical University, Xi’an, China; ^5^ Department of Radiation Oncology, Sun Yat-Sen University Cancer Center, Guangzhou, China

**Keywords:** non-small cell lung cancer, brain metastases, N stage, prognostic factor, grade prognostic assessment

## Abstract

**Background:**

Grade prognostic assessment (GPA) is widely used to evaluate the prognosis of non-small cell lung cancer (NSCLC) patients with brain metastases (BMs). This study aimed to investigate whether lymph node status (LNS) could be included as one of the GPA variables for NSCLC with BMs.

**Methods:**

Overall, 586 patients with NSCLC and BMs were retrospectively analyzed. Overall survival stratified by LNS was analyzed using the Kaplan-Meier method. Multivariate analysis was also performed to identify independent prognostic factors using the Cox proportional hazards progression model. In the updated GPA index, prognostic factors and criteria of GPA score were weighted by effect magnitude relative risk (RR) and statistical significance.

**Results:**

In NSCLC patients with BMs, those with lymph node involvement had worse overall survival (mOS, 13.4 months vs. 25.9 months, P <0.001) than those without lymph node involvement. Multivariate analysis showed that LNS might be an independent prognostic factor (RR: 1.702, CI: 1.340–2.162, P <0.001). Finally, five prognostic factors including LNS, the age of the patient, Karnofsky performance status (KPS), the number of BMs, and extracranial metastases were enrolled in our novel GPA index. With the updated GPA index involving the N stage, survival analysis was also performed. Prognostic results were significantly different among these four subgroups (Class A vs. Class B, P=0.047; Class B vs. Class C, P<0.001; Class C vs. Class D, P=0.007).

**Conclusions:**

These results indicate that LNS might be an indispensable prognostic factor in NSCLC with BM. The novel GPA model involving the N stage could provide more reliable evidence to estimate the survival of NSCLC patients with BMs.

## Introduction

About 25%–40% of non-small cell lung cancer (NSCLC) patients experienced brain metastases (BMs) during their disease course (1, 2). Many traditional therapeutic modalities, including surgical resection, stereotactic radiosurgery (SRS), whole-brain radiotherapy (WBRT), and chemotherapy, are available to oncology clinicians for treating NSCLCs with BMs. Unfortunately, the median overall survival remains poor, at only eight months (3, 4). Recently, the advent of targeted therapy and immunotherapy revolutionarily improve the survival of these patients depending on their molecular type and clinical characteristics (5–7). Therefore, it is vital to identify the factors affecting the prognosis of NSCLC. In clinical practice, grade prognostic assessment (GPA) has been widely used to evaluate the prognosis of NSCLC patients with BM and has provided evidence for clinicians to make treatment decisions. The original GPA index was composed of four prognostic factors: age of patients, Karnofsky performance status (KPS), number of BM, and extracranial metastases. Patients with BM were divided into four classes based on this index, with median survival ranging from 3.0 months to 14.8 months (8, 9). However, some studies have indicated that the number of BM might not be an independent prognostic variable in patients with BM (10). Moreover, NSCLC with BM is a systemic disease, and many factors affect its prognosis (11); therefore, it is necessary to update the original GPA index.

Lymph node status (LNS), both in clinical and pathological views, has been recognized as an indispensable prognostic factor for NSCLC. LNS also affects the choice of treatment modality for early stage NSCLC patients (12, 13). In the TNM classification, patients with distant metastasis were categorized as stage IV, regardless of N status (14, 15). One study indicated that LNS cannot be ignored even in stage IV patients. In that study, the author investigated whether LNS affected the prognosis of patients with M1a stage NSCLC (16). Survival analysis was performed in 39,731 patients with M1a disease from the Surveillance, Epidemiology, and End Results (SEER) database. Interestingly, the authors found that LNS was an independent prognostic factor for M1a patients, and similar results were obtained in all subgroups.

Patients with BM are also classified as M stage according to TNM classification, and some scattered data suggests that LNS might be related to BM. A study showed that there was no BM detected by MRI in N0 stage patients, but it was detected in 5.2% of N1 patients and 4.7% of N2 patients (17). Similarly, another study showed that patients with advanced N stage cancer (P=0.009) and diameter of lymph node >2.0 cm (P=0.027) might have a higher risk of experiencing BM during their disease course (18). However, no studies have reported the influence of LNS on the prognosis of NSCLC patients with BM.

Thus, our study was designed to identify whether LNS affects the prognosis of NSCLC patients with BM, and to determine whether the N stage involving GPA index was an important supplement to the original GPA in predicting the survival of NSCLC patients with BM.

## Materials and Methods

### Patient Selection

All patients who were first diagnosed with BM in NSCLC who were treated at Tangdu Hospital from 2003 to 2013 were eligible for enrollment in our study. The study was approved by the Review Board of the Air Force Medical University. The inclusion criteria were as follows: (1) histologically or cytologically diagnosed as NSCLC; (2) MRI or CT demonstrated the presence of BM; (3) able to be assessed for LNS by thoracic and cervical CT or PET/CT; and (4) able to supply adequate clinical information and available for follow-up.

### Evaluation of LNS

The N stage was defined according to the 8^th^ TNM classification (15): The N0 stage was defined as the absence of regional lymph node metastasis; N1 as metastasis in the ipsilateral peribronchial, perihilar, or intrapulmonary lymph nodes; N2 as metastasis in the ipsilateral mediastinal and/or subcarinal lymph nodes; and N3 as metastasis in the contralateral mediastinal, contralateral hilar, ipsilateral or contralateral scalene, or supraclavicular lymph nodes. Non-regional lymph node metastasis was defined as M stage. Lymph node involvement was defined as the shortest dimension of any lymph node ≥1.0 cm, according to CT scan or as diagnosed by PET/CT (19, 20). The LNS of the patients was independently reviewed by two radiologists.

### Statistical Analysis

The association between LNS and clinicopathologic factors was evaluated using the Chi-square test. Overall survival was defined as the time from diagnosis of BM to cancer-related death. We performed univariate and multivariate analyses using the Kaplan-Meier method and Cox proportional hazards progression model, respectively. A two-sided P-value <0.05 was considered statistically significant. In the updated GPA index, prognostic factors were weighted by effect magnitude relative risk (RR) and statistical significance. The scoring criteria for GPA were based on a previous study and the effect magnitude RR (9, 11). All analyses were performed using SPSS 22.0 software (IBM, Inc., Armonk, NY, USA).

## Results

### Patient Characteristics

We included 586 patients treated between 2003 and 2013 in our study. Among these patients, 156 patients underwent CT scanning, and three patients were confirmed by MRI to have BM after they received controversial negative CT results. The median age was 55 years (range, 23–80 years), prevalent histology was adenocarcinoma (87.5%), and epidermal growth factor receptor (EGFR) mutation status was detected in 109 patients (18.6%), including 57 patients of wild-type and 52 patients of mutant type. The remaining 477 patients did not undergo EGFR status testing. Among these patients, 201 received first-generation tyrosine kinase inhibitors (TKIs) therapy, such as gefitinib and erlotinib, administered orally. Other clinical-pathologic characteristics of the patients are listed in **Table 1**.

**Table 1:** Relationship between LNS and clinicopathological factors.

Variable	LNS^1^		P
	Positive	Negative	
Gender			0.925
Male	265	110	
Female	148	63	
Age			0.434
<70	372	160	
≥70	41	13	
Smoking status			0.057
Never	204	101	
Ever	209	72	
Histology			0.496
AC^2^	364	149	
NAC^3^	49	24	
EGFR mutation status			0.127
Wild	34	23	
Mutant	23	29	
KPS^4^			<0.001
<70	146	55	
70-80	220	75	
≥90	47	43	
Number of BM			0.020
≤3	266	129	
>3	147	44	
Size of BM^5^			0.070
<1.2cm	211	74	
≥1.2cm	202	99	
Extracraninal metastases			<0.001
No	162	102	
Yes	251	71	
Traditional GPA^6^ scores			0.005
Class A:0–1	127	33	
Class B:1.5–2	148	63	
Class C:2.5–3	114	57	
Class D:3.5–4	24	20	

^1^LNS, lymph node status; ^2^AC, adenocarcinoma; ^3^NAC, non-adenocarcinoma; ^4^KPS, Karnofsky performance status; ^5^size of BM, median diameter of the largest brain metastasis; ^6^GPA, graded prognostic assessment.

### Correlation Between LNS and Patient Baseline Characteristics

Among the 586 patients, 413 (70.5%) had lymph node involvement, including 136 patients diagnosed with N1 stage, 160 patients diagnosed with N2 stage, and 117 patients diagnosed with N3 stage. Patients with extracranial metastasis and higher GPA scores had a significantly higher rate of lymph node involvement (P <0.001 and P=0.005, respectively) than patients with no extracranial metastasis and lower GPA scores (**Table 1**).

### Comparison of Overall Survival Among Different Clinical N Stages

**Figure 1:**
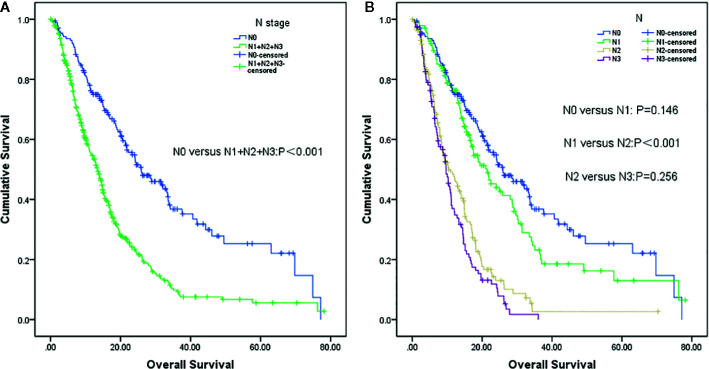
Survival analysis of non-small cell lung cancer (NSCLC) patients with brain metastases (BM) based on lymph node status (LNS). **(A)** Lymph node negative status (N0) vs. lymph node positive status (N1+N2+N3) (P<0.001). **(B)** N0 vs N1 (P=0,146), N1 vs. N2 (P<0.001) and N2 vs. N3 (P=0.256), respectively.

Patients with lymph node involvement had worse overall survival (mOS, 13.4 months vs. 25.9 months, P <0.001) than those without, as shown in **Figure 1A**. Further analysis revealed that patients in the N1 stage had better survival than those in the N2 stage, whereas there was no difference between N0 and N1 patients. A similar result was observed in N2 and N3 stage patients (N0 vs. N1, P=0.146; N1 vs. N2, P<0.001; and N2 vs. N3, P=0.256) (**Figure 1B**).

### Univariate and Multivariate Survival Analyses for the Updated GPA Model

**Figure 2:**
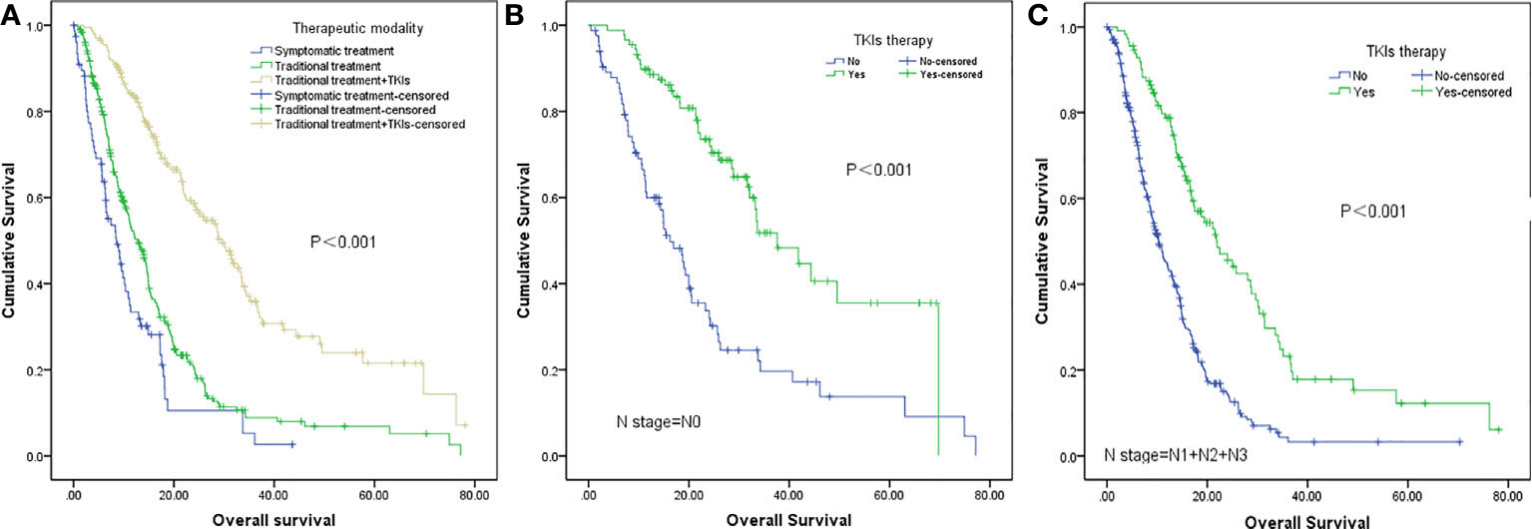
Survival analysis of non-small cell lung cancer (NSCLC) patients with brain metastases (BM) based on different therapeutic strategies. **(A)** Symptomatic treatment vs traditional treatment vs traditional treatment + TKIs (P<0.001). **(B)** TKIs therapy vs no TKIs therapy in patients with lymph node-negative (N0) status (P<0.001). **(C)** TKIs therapy vs no TKIs therapy in patients with lymph node-positive (N1+N2+N3) status (P<0.001).

The median overall survival was 15.4 months. Univariate analysis showed that the following factors were significantly correlated with overall survival: LNS (P <0.001), age (P=0.019), KPS (P <0.001), smoking status (P=0.008), number of BM (P=0.006), GPA (P <0.001), extracranial metastases (P <0.001), and therapeutic modality (P <0.001) (**Figure 2**), which are shown in **Table 2**. When the above significant variables were selected for inclusion in the multivariate analysis, LNS was found to be an independent prognostic factor in NSCLC patients with BM (RR: 1.702, CI: 1.340–2.162, P <0.001). The age of patients (RR: 1.581), KPS (RR: 1.845), extracranial metastases (RR: 1.519), and treatment modality (RR: 0.374) were shown to be prognostic factors by multivariate analysis (**Table 3**).

**Table 2:** Univariate analysis of overall survival of NSCLC patients with BM.

Variable	N	OS(median)	95%CI	P
	586	15.400	14.064–16.736	
Gender				0.808
Male	375	16.533	14.823–18.244	
Female	211	14.733	12.203–17.264	
Age				0.019
<70	532	16.267	14.783–17.751	
≥70	54	11.067	8.798–13.336	
Smoking status				0.008
Never	305	17.500	14.756–20.244	
Ever	281	14.700	12.892–16.508	
Histology				0.911
AC^1^	513	15.500	14.065–16.935	
NAC^2^	73	14.967	9.418–20.516	
EGFR mutation				0.087
Wild	57	25.933	18.351–33.516	
Mutant	52	35.100	30.127–40.073	
KPS^3^				<0.001
<70	201	10.700	8.313–13.087	
70–80	295	17.133	14.997–19.270	
≥90	90	34.300	23.356–45.244	
Number of BM				0.006
≤3	395	16.767	14.779–18.755	
>3	191	13.300	11.189–15.411	
Size of BM^4^				0.082
<1.2 cm	285	16.933	14.275–19.592	
≥1.2 cm	301	14.933	13.535–16.332	
Extracraninal metastases				<0.001
No	264	19.933	16.011–23.8565	
Yes	322	13.267	11.415–15.118	
N stage				<0.001
N0	173	25.900	18.811–32.989	
N1	136	21.500	16.231–26.769	0.146
N2	160	10.367	7.083–13.651	<0.001
N3	117	9.700	8.351–11.04	0.256
Traditional GPA^5^ scores				<0.001
Class A:0–1	160	10.633	8.939–12.327	
Class B:1.5–2	211	16.267	14.196–18.337	<0.001
Class C:2.5–3	171	118.833	16.210–21.457	0.347
Class D:3.5–4	44	74.900	23.568–126.232	<0.001
Treatment modality				<0.001
Symptomatic treatment^6^	78	8.567	5.675–11.459	
Conventional therapy^7^	307	12.800	10.875–14.725	
TKIs therapy^8^	201	29.667	25.318–34.015	

^1^AC, adenocarcinoma; ^2^NAC, non-adenocarcinoma; ^3^KPSm Karnofsky performance status; ^4^size of BM, median diameter of the largest brain metastasis; ^5^GPA, graded prognostic assessment; ^6^Symptomatic treatment, reducing intracranial pressure treatment: Mannitol 125mg/time, twice every day, intravenous drip; ^7^Conventional therapy, systemic chemotherapy plus local treatment; ^8^TKIs therapy, first generation of tyrosine kinase inhibitors (TKIs): gefitinib or erlotinib.

**Table 3:** Results of multivariate analysis for overall survival by Cox regression model.

Variable	RR	95%CI	P
Age (≥70years vs <70years)	1.581	1.137–2.199	0.006
KPS^1^ (≥90/70–80/<70)	1.845	1.565–2.175	<0.001
Extracranial metastases (Yes vs No)	1.519	1.229–1.879	<0.001
LNS (N positive vs N negative)	1.702	1.340–2.162	<0.001
Treatment modality (TKIs^2^ vs Conventional therapy^3^)	0.374	0.297–0.472	<0.001

^1^KPS, Karnofsky performance status; ^2^TKIs therapy: first generation of tyrosine kinase inhibitors (TKIs), gefitinib and erlotinib; ^3^Conventional therapy, symptomatic or systemic treatment.

**Figure 3:**
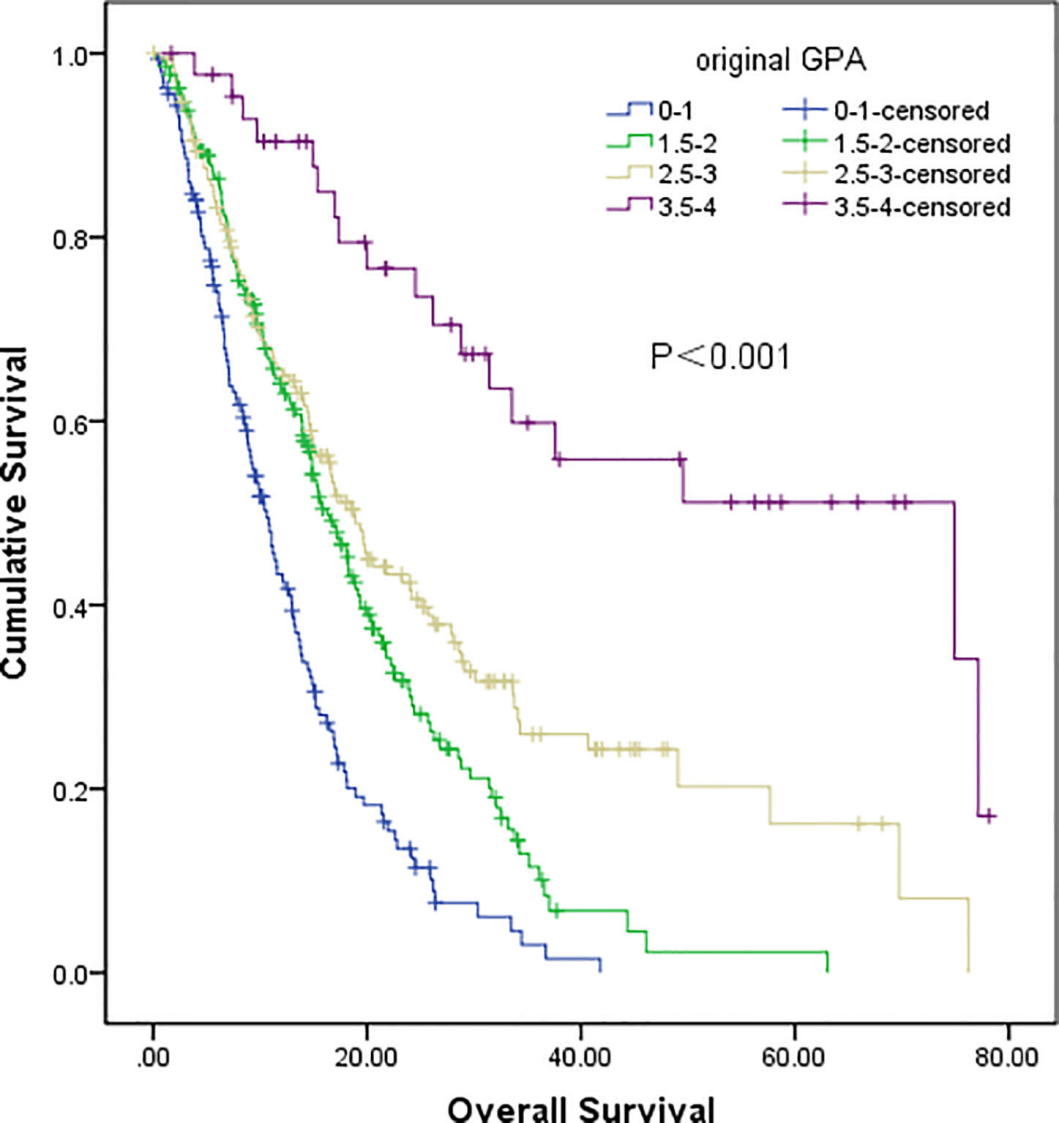
Survival analysis of non-small cell lung cancer (NSCLC) patients with brain metastases (BM) based on original GPA (Class A vs Class B, P<0.001; Class B vs Class C, P=0.347; Class C vs Class D, P<0.001).

The original GPA index consisted of four variables, including age, KPS, number of BM, and extracranial metastases (**Table 4**). We demonstrated that traditional GPA could be used to evaluate the prognosis of BM patients, especially when selecting the patients with the best or worse prognoses (Class A vs. Class B, P<0.001; Class C vs. Class D, P<0.001), but there was no significant difference between Class B and Class C (P=0.347) (**Figure 3**, **Table 2**). From our data, age, KPS, and extracranial metastases, were proven to be independent prognostic factors (**Table 3**).

**Table 4:** Criteria of traditional graded prognostic assessment (GPA) for NSCLC with brain metastases.

Variable		Traditional GPA criteria	
	0	0.5	1.0
Age(years)	>60	50–60	<50
KPS^1^	<70	70–80	>90
Extracrainial metastases	Yes	NA^2^	No
Number of BM	>3	2–3	1

^1^KPS, Karnofsky performance status; ^2^NA, not applicable.

**Figure 4:**
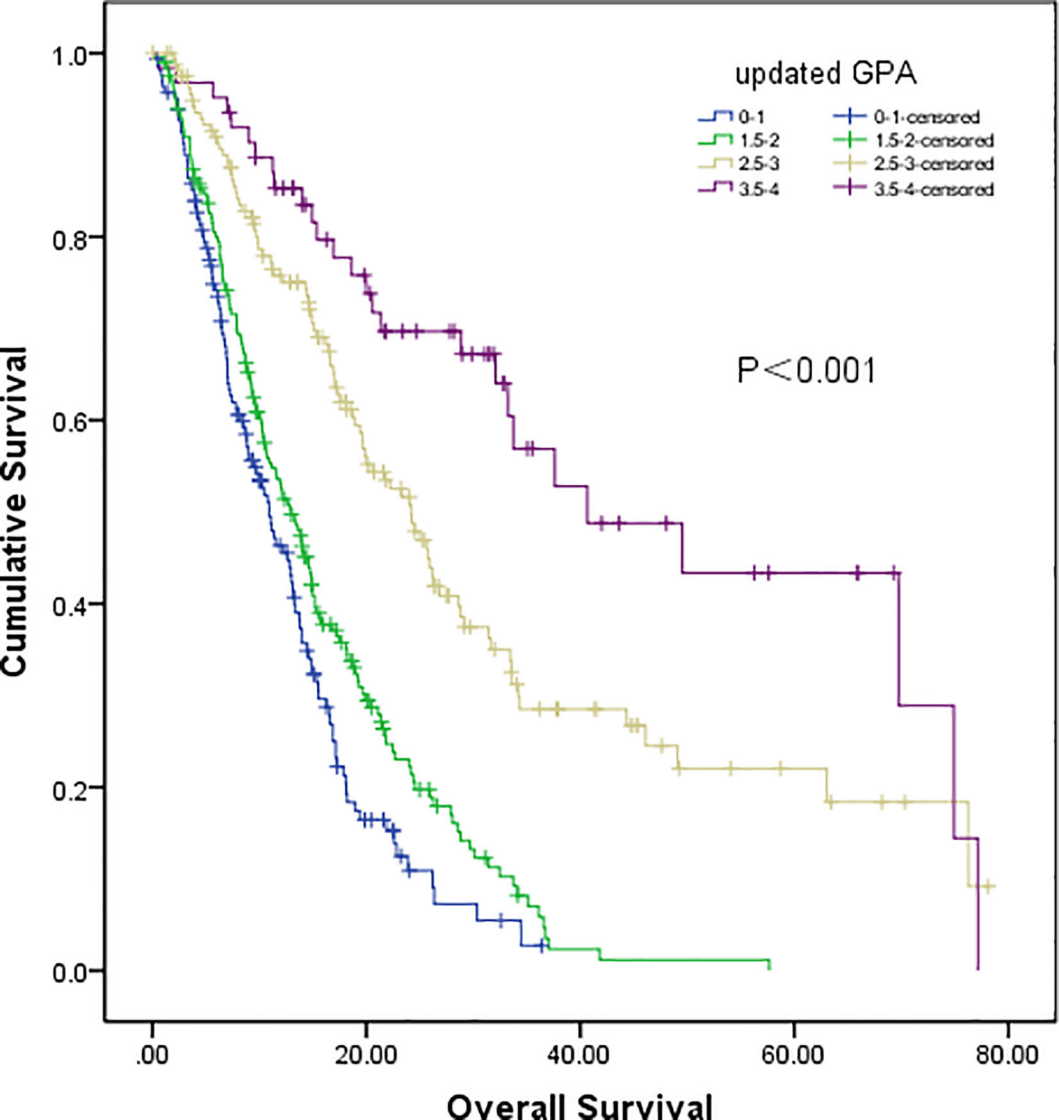
Survival analysis of non-small cell lung cancer (NSCLC) patients with brain metastases (BM) based on updated GPA (Class A vs Class B, P=0.047; Class B vs Class C, P<0.001; Class C vs Class D, P=0.007).

In our updated GPA index, prognostic factors were selected on the basis of the effect magnitude relative risk (RR) and statistical significance. The number of BMs in our novel GPA model was still selected based on previous studies and the univariate analysis results in our study. Finally, five prognostic factors, including LNS, age of patient, KPS, number of BM, and extracranial metastases, were enrolled in our novel GPA index. The scoring criteria were based on a previous study and RR, so extracranial metastases, KPS, and LNS were given a maximum score of 1.0, and the remaining two variables were given a maximum score of 0.5, for a total score of 4.0 (**Table 5**). With the updated GPA index, survival analysis was also performed. The prognostic results were significantly different among these four subgroups (Class A vs. Class B, P=0.047; Class B vs. Class C, P<0.001; Class C vs. Class D, P=0.007), as shown in **Figure 4** and **Table 6**.

**Table 5:** Updated GPA based on LNS for NSCLC with brain metastases.

Variable		Novel GPA criteria	
	0	0.5	1.0
Age(years)	≥55	<55	NA
KPS^1^	<70	70-80	>90
Extracrainial metastases	Yes	NA^2^	No
Number of BM	≥3	<3	NA
LNS^3^	N(+)	NA	N(-)

^1^KPS, Karnofsky performance status; ^2^NA, not applicable; ^3^LNS, lymph node status.

**Table 6:** Survival analysis base on GPA index in NSCLC patients with BM.

Variable	N	OS(median)	95%CI	P
	586	15.400	14.064–16.736	
Traditional GPA^1^ scores				<0.001
Class A:0–1	160	10.633	8.939–12.327	
Class B:1.5–2	211	16.267	14.196–18.337	<0.001
Class C:2.5–3	171	118.833	16.210–21.457	0.347
Class D:3.5–4	44	74.900	23.568–126.232	<0.001
Updated LNS^2^ based GPA scores				<0.001
Class A:0-1	220	10.933	8.174–13.693	
Class B:1.5-2	204	12.967	10.660–15.273	0.047
Class C:2.5-3	128	24.167	19.276–29.057	<0.001
Class D:3.5-4	34	40.667	21.190–60.143	0.007

^1^GPA, graded prognostic assessment; ^2^LNS, lymph node status.

## Discussion

We classified NSCLC patients with distant metastasis as stage IV. The 8^th^ edition of the TNM stage classification divided patients into M1a, M1b, and M1c based on the heterogeneity of treatment and prognosis. Patients with NSCLC and BM should be defined as in stage M1b or M1c, according to the new staging version (14). A previous study demonstrated that LNS could provide prognostic information for patients with NSCLC with intrathoracic metastasis (M1a stage) (16). However, there are few related studies on NSCLC with BM. This study strongly suggests that LNS is an indispensable prognostic factor for patients with BM.

The prognostic value of LNS for patients with stage IV NSCLC has not been extensively studied. Iida et al. (21) reported that LNS was an independent prognostic factor for patients with pleural dissemination (M1a stage). Another study included 39,731 patients with M1a disease, and subsequent survival analysis showed that M1a patients without lymph node metastasis had the best survival, followed by patients in the N1 stage; however, there was no significant difference between N2 and N3 stage patients (N0 vs N1, P<0.001; N1 vs. N2, P<0.001; and N2 vs. N3, P=0.478) (16). Similar to the above two studies, our results strongly suggest that the N0 stage was an independent predictor of better survival for NSCLC patients with BM.

The original GPA index, including four variables (age, KPS, number of BM, and extracranial metastases), was widely used to predict the prognosis of NSCLC patients with BM. The four factors of the GPA index could predict the prognosis of these patients (8); however, these results have not been consistently observed in other studies, especially for the number of BM (10, 22). Indeed, although the number of BM (n≤ 3) was not an independent prognostic factor in our study, we still included it in both the original and updated GPA scoring systems. Moreover, we attempted to evaluate the effect of the updated GPA index involving the N stage; hence, the original GPA index was verified equally, which showed that the original GPA could significantly identify the prognosis of NSCLC patients with BM, especially for selecting patients with the best or worst prognoses (Class A and Class D). However, it failed to stratify the difference between the patients of Class B and Class C. According to the updated GPA index involving the N stage, the prognostic results were significantly different among these four subgroups.

Moreover, with extensive research on the mechanism of driver genes in NSCLC, the GPA index has continually been updated to precisely distinguish the prognosis of NSCLC patients with BM. Balasubramanian et al. reported that in NSCLC patients with BM, the median overall survival for patients with EGFR/ALK mutations was longer than that of wild-type patients (P=0.028) (10). Similarly, a meta-analysis that included 4,373 patients from 18 studies was designed to evaluate the relationship between EGFR mutation status and overall survival of patients with NSCLC with BM. The results also confirmed that EGFR mutation status is an important prognostic factor for NSCLC with BM (23). Therefore, a new GPA index was built using the driver genes and molecular alterations of NSCLC patients with BM (11). In Sperduto’s research, significant prognostic factors included the classical four factors (age, KPS, extracranial metastases, and number of BM) and two molecular factors: EGFR and ALK mutant status, which frequently occurred in lung adenocarcinoma. The median OS for the whole cohort in this study setted in US was 12.0 months, and those adenocarcinomas with Lung-molGPA scores of 3.5 to 4.0 had a median survival of nearly 4 years, despite the lower prevalence of drive gene mutation in Caucasian patients compared with East Asian patients. It indicate that driver gene alteration involving GPA scores is a meaningful tool that may facilitate clinical decision-making of NSCLC patient with BM. Similar data were also found in our study (EGFR mutant vs EGFR Wild), although only 109 patients underwent EGFR mutant testing and the other drive genes detections were absent in our study. So a large sample and molecular typing based GPA model is going to be designed in Chinese patients in our future study.

The usability and popularization of a GPA model mostly depends on whether it can widely cover all patients with NSCLC with BM; therefore, the enrolled patients should include patients receiving traditional intervention as well as targeted treatment. In the present study, 201 patients received targeted therapy. The IPASS study (the Iressa Pan-Asia study) is a landmark study on NSCLC, demonstrating the superiority of TKI as a first-line treatment compared to chemotherapy for EGFR mutant patients with respect to progression-free survival (PFS) (24). Moreover, data from the CTONG-0803 trial (25), a phase II, open-label prospective study that evaluated the efficiency of erlotinib in NSCLC with BM, showed that EGFR-positive patients had longer PFS than those with EGFR-negative patients (15.2 months vs. 4.4 months, P = 0.02). In our previous study, we found that TKI treatment was beneficial for BM patients in terms of both PFS and overall survival (OS), independent of the EGFR mutation status (26). Our data confirmed again that TKI therapy could prolong the overall survival of all BM patients, regardless of the N stage.

Finally, it is controversial whether NSCLC patients with BM could benefit from thoracic therapy. Aggressive thoracic therapy (ATT) was defined as resection of the primary disease or chemoradiotherapy in which the total radiation dose exceeded 45 Gy. Gray et al. (27) reported that patients with synchronous and brain-only oligometastatic disease who received ATT had a longer overall survival (P < 0.001), and this survival benefit was also found in patients with stage III disease (P = 0.004). This suggests that thoracic therapy might prolong the survival of BM patients, especially for oligometastatic patients.

Some limitations of this study should be mentioned. The first is that LNS was primarily evaluated by imaging technology (CT or PET/CT), and only a small percentage of patients underwent mediastinoscopy examination. Dales et al. (19) reported that the sensitivity and specificity of CT for mediastinal lymph node diagnosis were 78% and 79%, respectively. Even with PET/CT, the sensitivity and specificity were 85% and 90%, respectively (20). Moreover, the therapeutic schedule of patients was not uniform; only 201 patients received TKI therapy for different lines, and none had been treated with osimertinib, a third-generation TKI, which is widely used in the treatment of BM patients harboring EGFR mutations (28). Finally, data on mediastinal radiotherapy for patients with mediastinal lymph node involvement were limited (29). A study on WBRT/SRS combined with intrathoracic radiotherapy for BM patients positive for lymph node involvement should be designed in the future.

In conclusion, our study provides preliminary evidence that LNS might be an indispensable prognostic factor in NSCLC with BM, and the novel grade prognostic assessment (GPA) model involved in the N stage could provide more information to predict survival.

## Data Availability Statement

The raw data supporting the conclusions of this article will be made available by the authors, without undue reservation.

## Ethics Statement

The studies involving human participants were reviewed and approved by Tangdu Hospital of Air Force Medical University. The patients/participants provided their written informed consent to participate in this study.

## Author Contributions

TJ, LZ, and LC participated in study design and study conception. JZ, WPW, SX, HW, and ZZ performed data analysis. JZ, WPW, SX, CJ, YX, WCW, MW, and XW recruited patients. JZ, WPW, SX, and LC drafted the manuscript. All authors contributed to the article and approved the submitted version.

## Funding

This study was supported by grants from the Wu Jieping Medical Foundation (320. 6750. 17527) and the Provincial Key R&D Program of Shaanxi (2017ZDCXL-SF-01-04-01).

## Conflict of Interest

The authors declare that the research was conducted in the absence of any commercial or financial relationships that could be construed as a potential conflict of interest.
